# It takes two (seconds): decreasing encoding time for two-choice functional near-infrared spectroscopy brain–computer interface communication

**DOI:** 10.1117/1.NPh.10.4.045005

**Published:** 2023-11-02

**Authors:** Anna Vorreuther, Lisa Bastian, Amaia Benitez Andonegui, Danielle Evenblij, Lars Riecke, Michael Lührs, Bettina Sorger

**Affiliations:** a Maastricht University, Department of Cognitive Neuroscience, Maastricht, The Netherlands; b University of Stuttgart, Institute of Human Factors and Technology Management IAT, Applied Neurocognitive Systems, Stuttgart, Germany; c University of Tübingen, Institute of Medical Psychology and Behavioral Neurobiology, Tübingen, Germany; d International Max Planck Research School, Graduate Training Centre of Neuroscience, Tübingen, Germany; e NIH, MEG Core Facility National Institute of Mental Health, Bethesda, Maryland, United States; f Brain Innovation B.V., Research Department, Maastricht, The Netherlands

**Keywords:** motor disability, “locked-in” syndrome, brain-based communication, brain–computer interface, fNIRS, mental imagery, online data analysis

## Abstract

**Significance:**

Brain–computer interfaces (BCIs) can provide severely motor-impaired patients with a motor-independent communication channel. Functional near-infrared spectroscopy (fNIRS) constitutes a promising BCI-input modality given its high mobility, safety, user comfort, cost-efficiency, and relatively low motion sensitivity.

**Aim:**

The present study aimed at developing an efficient and convenient two-choice fNIRS communication BCI by implementing a relatively short encoding time (2 s), considerably increasing communication speed, and decreasing the cognitive load of BCI users.

**Approach:**

To encode binary answers to 10 biographical questions, 10 healthy adults repeatedly performed a combined motor-speech imagery task within 2 different time windows guided by auditory instructions. Each answer-encoding run consisted of 10 trials. Answers were decoded during the ongoing experiment from the time course of the individually identified most-informative fNIRS channel-by-chromophore combination.

**Results:**

The answers of participants were decoded online with an accuracy of 85.8% (run-based group mean). Post-hoc analysis yielded an average single-trial accuracy of 68.1%. Analysis of the effect of number of trial repetitions showed that the best information-transfer rate could be obtained by combining four encoding trials.

**Conclusions:**

The study demonstrates that an encoding time as short as 2 s can enable immediate, efficient, and convenient fNIRS-BCI communication.

## 1 Introduction

Individuals with severe motor disabilities caused by diseases, such as late-stage amyotrophic lateral sclerosis may experience a state known as “locked-in” syndrome. They remain conscious and aware of their surroundings while experiencing complete or near-complete motor paralysis. Thus, natural means of communication with these patients might become impossible albeit essential in daily life and for treatment. With the aim to establish alternative brain-based means of communication for these individuals, brain–computer interfaces (BCIs) have been suggested and developed. BCIs enable users to communicate via intentionally evoked brain signals recorded through various functional-neuroimaging methods.^1^^–^^17^

Electroencephalography (EEG) has been the most widely used input modality for BCIs due to its high temporal resolution and general availability. However, not all individuals can successfully use an EEG-based BCI:^18^ some individuals may produce excessive motion artifacts that mask brain activity necessary to control a particular BCI; or they cannot generate strong enough brain activity that is detectable on the scalp.^19^ Therefore, hemodynamic neuroimaging methods, such as functional magnetic resonance imaging (fMRI) and functional near-infrared spectroscopy (fNIRS), have been additionally explored in the context of BCIs.^20^ These non-invasive methods measure brain activity indirectly through the neurovascular response.^21^ Especially fNIRS has recently received increased attention as a BCI-input modality as it is safe, quick to set up, easy to operate, its application is almost silent, and recordings are feasible even in natural body postures. Moreover, it is a portable and relatively inexpensive method. Thus, it could eventually be used in clinical routine or ultimately at home of potential users.^22^^–^^24^

In numerous studies with healthy participants (e.g., Refs. 2, 9, 22, and 25–36) and patients (e.g., Refs. 1, 15, 37, and 38), different methodological aspects of motor-independent fNIRS-based communication have been investigated. Generally, for motor-independent communication, covert (mental) tasks are used to elicit voluntary modulation of brain activation. While motor-imagery tasks^2^^,^^9^^,^^25^^–^^27^^,^^29^^,^^30^^,^^32^^,^^37^ and mental-calculation tasks^1^^,^^22^^,^^28^^,^^29^^,^^31^^,^^32^^,^^34^^,^^36^^,^^38^ are used most often, additional strategies, such as mental singing/music imagery,^1^^,^^31^^,^^33^^,^^34^ speech,^15^^,^^35^ or spatial navigation,^25^ have been shown to work effectively as well. Mostly, encoding instructions are provided via auditory^1^^,^^15^^,^^25^^–^^27^^,^^37^ or visual cues.^2^^,^^9^^,^^28^^–^^36^^,^^38^ Recently, also tactile guidance has been successfully implemented.^27^ To detect voluntary modulation of brain activation, temporal,^1^^,^^9^^,^^22^^,^^26^^–^^28^^,^^30^^,^^31^^,^^37^^,^^38^ spatial,^2^^,^^15^^,^^29^^,^^32^^–^^34^ or both signal features combined^25^^,^^36^ have been utilized. The number of encoding options is so far mostly limited to binary choices,^1^^,^^2^^,^^9^^,^^15^^,^^22^^,^^25^^,^^26^^,^^28^^,^^32^^–^^35^^,^^37^^,^^39^^–^^41^ nevertheless multiple-choice paradigms^27^^,^^29^^,^^31^^,^^36^^,^^38^ allowing up to six-class classification^30^ have been demonstrated to be possible as well. Depending on the fNIRS-signal features used for decoding, univariate^9^^,^^26^^–^^28^^,^^30^^,^^37^ or multivariate analysis strategies^2^^,^^15^^,^^25^^,^^29^^,^^31^^,^^38^ have been applied. FNIRS-BCI studies are usually conducted in a laboratory environment. However, lately the potential of fNIRS as an in-the-field BCI technique has been demonstrated when participants successfully communicated multiple-choice answers in a cafeteria.^27^ All these studies show that the fNIRS-BCI approach is very promising. One unfavorable aspect, however, is the limited temporal resolution inherent to all hemodynamic neuroimaging methods. As a result, implemented encoding times have been relatively long, ranging from 6 s^30^^,^^38^ up to 30 s^37^ while most studies use 10 to 20 s.^1^^,^^2^^,^^9^^,^^22^^,^^25^^,^^27^^–^^29^^,^^31^ This has limited communication speed and required relatively high cognitive demand and endurance from BCI users.

The current study aimed at increasing the speed of fNIRS-based communication using a shortened encoding time of 2 s. A temporal encoding approach was implemented: healthy participants freely communicated their binary answers by performing a mental-imagery task at temporally distinct encoding periods. To activate a large portion of the cortex and therewith increase the chance of evoking sufficient brain activation, a combined motor-speech imagery task was used. The study shows that an encoding time as short as 2 s is indeed feasible, making fNIRS-BCI communication more efficient and less cognitively demanding.

## 2 Methods

### 2.1 Participants

The experimental procedure conformed to the Declaration of Helsinki and was approved by the local ethics committee [Ethics Review Committee Psychology and Neuroscience (ERCPN)]. Ten healthy individuals [10 females, 9 right-handed, age = 26.0 ± 7.2 years (mean ± SD), all with reportedly normal hearing; see Table 1] were externally recruited or were recruited from students and staff members of the Faculty of Psychology and Neuroscience at Maastricht University. Written informed consent was acquired prior to the experiment. Participants were compensated financially for their participation (7.50 €/h in form of vouchers).

**Table 1:** Participant demographics.

Participant	Age (years)	Gender	Handedness	FNIRS experience	Cap size (cm)
P01	25	Female	Right	No	56
P02	22	Female	Right	No	58
P03	47	Female	Right	Yes	56
P04	24	Female	Right	Yes	56
P05	26	Female	Right	Yes	56
P06	21	Female	Left	No	58
P07	21	Female	Right	No	56
P08	24	Female	Right	No	58
P09	21	Female	Right	No	58
P10	31	Female	Right	Yes	56

Note. Participant demographics are displayed.

### 2.2 General Procedure

**Fig. 1:**
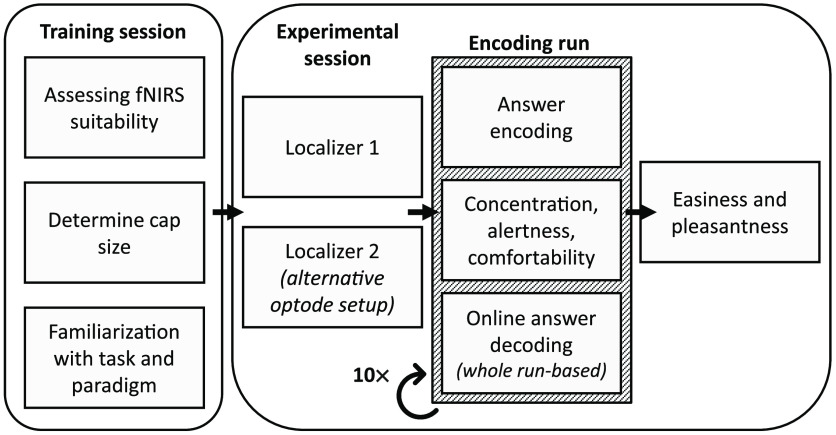
General procedure. Each participant underwent a training session (day 1) comprising of assessing fNIRS suitability, determining the correct cap size, and familiarization with the mental-imagery task and encoding paradigm. In the experimental session (day 2) two localizers were performed to determine a COI followed by 10 encoding runs to encode answers and decode the corresponding signal online. Subjective ratings were acquired after each run and finally the mental-imagery task was rated at the end of the session.

The study was composed of a training session and the fNIRS-BCI session (see Fig. 1). The training session entailed a training of the mental imagery task and familiarization with the communication paradigm. To evoke a measurable signal, a combined motor-speech imagery task was used. Participants were asked to imagine checking off a list with their right hand while saying “check” or “correct” simultaneously for ∼2  s. Each participant was instructed on how to perform the mental task and trained to encode answers until they felt comfortable in doing so (for ∼15  min). Additionally, participants’ head circumference was measured to select an appropriate cap size. Cap sizes used in this experiment ranged from 56 to 58 cm (see Table 1). After training, participants chose 10 unobtrusive binary questions to be answered during the BCI session from a list of 45 possible questions (see Appendix 1 in the Supplementary Material). To ensure an equal distribution of both answer options regardless of choice, each question was to be answered truthfully first before also encoding the opposite answer. Finally, to quantify participants’ suitability for fNIRS measurements, a suitability score was determined by means of an in-house questionnaire (see Appendix 2 in the Supplementary Material) completed prior to the measurements.^25^

During the second part of the study, the fNIRS-communication paradigm was tested. In the beginning, participants were seated in a sound-attenuating and darkened room. Nasion-inion distance was measured to ensure correct positioning of the cap. FNIRS optodes were additionally covered by a black cap to prevent influence of external light on the measurements. Finally, the room was equipped with a microphone and speakers to enable communication between participant and researcher and to play recorded auditory instructions given during the experiment. Participants were instructed to get into a comfortable position and to relax their jaw and facial muscles during the session.

The measurements commenced with two localizer runs to determine the most informative channel [channel of interest (COI)]. After the first localizer, participants were able to take a short break during which the channel setup was changed for the second localizer. Thus, each participant underwent two localizer runs with a different setup per run. To counterbalance possible effects of fatigue in the setup choice, half the participants started with one setup and the remaining half with the other one. Following the localizers, 10 encoding runs (1 per question) were performed. For encoding runs, the optode setup, including the most informative channel was chosen for each participant. In between runs, participants were allowed to take a short break, adjust posture, and drink water. After each run, they were asked to rate their current comfortability, concentration, and alertness on a scale between 1 (not at all) and 10 (very). After the final run, participants were asked to rate the easiness and pleasantness of the mental task.

### 2.3 Communication Paradigm

**Fig. 2:**
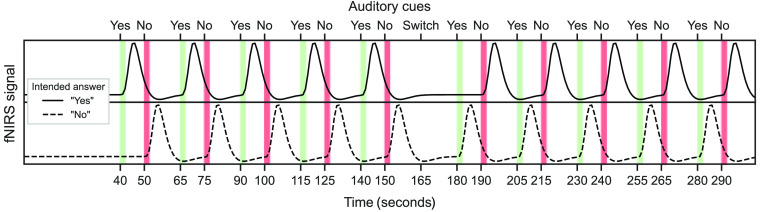
Answer encoding scheme. The figure shows the two possible answer-encoding scenarios for both a “yes” (top) and “no” (bottom) answer. Expected oxyhemoglobin (HbO) time courses (black curves) in mental imagery-related brain regions are displayed. When participants intended to encode “yes,” they performed combined motor-speech imagery causing HbO to rise during the encoding time span for “yes” (2s; green bars). When participants wished to encode “no,” they performed combined motor-speech imagery causing HbO to rise during the encoding time span for “no” (2s; red bars). Participants encoded the same answer five times per run before a designated switch (auditory cue “switch”) indicated to encode the opposite answer for five times again.

Participants answered binary questions by performing a mental-imagery task for ∼2  s at different points in time to indicate the answer. Simple auditory instructions, i.e., concise spoken commands (“yes,” “no,” and “switch”) informed participants of the start of each encoding window. Note that no explicit instruction to stop the mental imagery was required as participants had been trained to perform the task for ∼2  s. Instructions and corresponding time point triggers were implemented using the stimulation software NIRStim 4.0 (NIRx Medizintechnik GmbH, Berlin, Germany). Each run consisted of a 40 s baseline [to calculate oxyhemoglobin (HbO) and deoxyhemoglobin (HbR)] concentrations preceding 10 trials in which a question was answered 10 times in total (5:08 min/run; see Fig. 2). Each trial consisted of a 5-s pre-window, a 2-s window for one encoding option (cued by auditory instructions) followed by 8 s resting time before the second 2-s window (cued by auditory instructions), and then a 10-s post-window. In the first five trials, answers should be given truthfully and in the last five the opposite answer should be encoded. An auditory cue “switch” instructed participants when to change their encoded answer. This ensured that each answer option was encoded equally often independent of a participant’s choice of questions and answers. To indicate the answer, the participant had to perform the mental imagery task for about 2 s at the respective time of a trial. Each trial was initiated by the first auditory cue for indicating “yes.” If the participant wished to answer “yes,” they then performed the 2-s mental imagery. 10 s after the first auditory cue a second cue for “no” followed. To indicate the second answer option, the participant performed the task in the respective time window.

### 2.4 FNIRS Suitability Questionnaire

Because fNIRS constitutes an optical neuroimaging method, its signal quality can be crucially impacted by the presence of certain physical features of participants, such as hair characteristics (e.g., color, thickness, and density^42^^–^^47^), skin pigmentation,^47^ and head size (inter-optode distance and skull thickness variation affects light absorption). To quantify participants’ suitability for fNIRS measurements, a suitability score was determined by means of an in-house questionnaire (see Appendix 2 in the Supplementary Material) completed prior to the measurements.^25^ The physical features of interest were rated on a scale ranging from 0 to 4 regarding their risk to absorb NIR light. Individual ratings were summed up to a total score (possible scoring range: 1 to 21). A low fNIRS-suitability score has been associated with improved signal quality.^25^

### 2.5 Data Acquisition

Hemodynamic brain activity was measured with an NIRScout-816 system (NIRx Medizintechnik GmbH, Berlin, Germany). Eight detector and nine source optodes (small light emitting diodes were used to produce light at wavelengths of both 760 and 850 nm. Recorded signals were sampled at a rate of 6.94 Hz. Optodes were positioned according to the international 10 to 20 EEG system. The optodes always covered the left-hemispheric fronto-temporo-parietal cortex for optimal coverage of brain regions activated during the performance of the combined motor-speech imagery.^48^

**Fig. 3:**
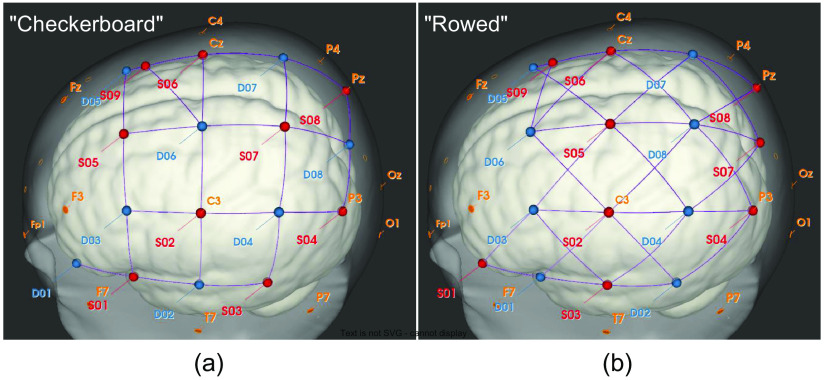
3D view of implemented fNIRS optode setups. “Checkerboard” (a) and “rowed” (b) setup were used during the initial localizer procedure. Light absorption was measured using nine sources (S; red) and eight detectors (D; blue) placed over the left-hemispheric (pre)motor and speech-related regions. For the “checkerboard” setup, sources were positioned on FC5 (1), C3 (2), CP5 (3), P3 (4), FC1 (5), Cz (6), CP1 (7), Pz (8), and 1 cm posterior of FCz (9; SDC source) while detectors were placed on F5 (1), C5 (2), FC3 (3), CP3 (4), FCz (5), C1 (6), CPz (7), and P1 (8). The average channel distance was 38.26 ± 3.5 mm (not including the SDC). For the “rowed” setup, sources were positioned on F5 (1), C3 (2), C5 (3), P3 (4), C1 (5), Cz (6), P1 (7), Pz (8), and 1 cm posterior of FCz (9; SDC source) while detectors were placed on FC5 (1), CP5 (2), FC3 (3), CP3 (4), FCz (5), FC1 (6), CPz (7), and CP1 (8). The average channel distance was 47.04±8.4  mm (not including the SDC). In total, the “checkerboard” setup contained 25 channels and the “rowed” setup contained 32 channels, including an SDC (S9-D5). The 3D representation was created with NIRSite v1.0 software (NIRx Medizintechnik GmbH, Berlin, Germany).

To increase the chance of defining a promising channel-by-chromophore combination for each participant for the subsequent encoding runs, two alternative setups were used in two initial localizer runs. For one setup, optodes were positioned in a checkerboard pattern, interleaving sources with detectors. For the other setup, positions of sources and detectors were changed thereby forming a rowed placement with rows of sources alternating with rows of detectors. Thus, one setup partially covered those cortical regions that were not covered by the alternative one (see Fig. 3).

A custom-build short-distance channel (SDC) was created by placing source S9 as close as the optodes would allow (∼10  mm away) to detector D5 on the same sagittal plane that connects D5 to source S6 (see Fig. 3). The mid-sagittal sinus covered by the added SDC and other large vascular structures^49^ have been shown to correlate with low frequency oscillations and cardiac signals.^50^ The SDC was used in both setups to acquire these signal influences and thereby account for the (extracerebral) physiological noise in the region covered by the setups.^30^ In total, the setups contained 25 (“checkerboard”) and 32 (“rowed”) direct-neighbor channels including the SDC, respectively.

### 2.6 Localizer Runs

Two localizer runs were performed for each participant to assess the strength of their hemodynamic responses and determine a channel most sensitive to the answer decoding. Each run consisted of 20 trials of 2 s each during which participants were instructed to encode “yes” for the first half of the trials followed by encoding “no” for the subsequent trials. An auditory spoken cue “switch” indicated to the participants when to alter their answer encoding. After the first localizer participants were able to take a break during which the channel setup was changed to another one for the second localizer. Thus, each participant underwent two localizer runs with a different setup per run. The first participant tested (here referenced as P07) performed 30 trials in total instead of 20 during the localizer but did not differ in number of trials during subsequent encoding runs.

### 2.7 Data Analysis

For data analysis, the software Turbo-Satori (v.1.2.8, Brain Innovation B. V., Maastricht, The Netherlands) was used.^51^ Measured time course values were converted to HbO and HbR values using the modified Beer–Lambert law. Moving-average high-pass and low-pass filters were applied (high-pass cut-off: 0.010 Hz, filter order: 1; low-pass cut-off: 0.250 Hz, filter order: 2). Data were analyzed online (i.e., in real-time) and offline using univariate general linear model (GLM) analysis.

Selection of an ideal channel and Hb-type for each participant was based on the localizer data. Specifically, predictors for each answer option were convolved with a standard hemodynamic response function (HRF; double Gamma function, the onset of response and undershoot was 6 and 16 s, respectively, dispersion 1 s, response to undershot ratio 6). A single most informative COI and corresponding most informative Hb-type was determined based on the chromophore and channel that led to the highest t-statistic of the active encoding versus rest contrast across localizer runs. Further, the SDC time course was used as a confound predictor to regress out noise. Localizer data were additionally used to create topographical maps of HbO and HbR activation changes obtained for a group fixed effects analysis (with n=10, Bonferroni-corrected p<0.01) during combined motor-speech imagery task for the setups using Satori (version 1.8). During answer-encoding runs, the encoded answer per run/question was decoded online by fitting a GLM to all 10 trials in each run. Two answer predictors were each convolved with an HRF to model all 10 trials. T-values for each predictor were determined based on the previously chosen COI and corresponding chromophore. The answer that led to the highest t-estimate of the contrast “yes” versus “no” was considered the selected option. From these answers, a whole-run accuracy was acquired for each participant by dividing the correctly encoded number of runs by the total number of performed runs. In addition, the SDC time course was used as confound predictor again. Subsequently, the data of the encoding runs of each participant were analyzed using offline GLM analysis to calculate a single-trial (ST) accuracy per participant. Mean accuracies were evaluated in a confusion matrix per participant using a $\chi^{2}$ test to identify participants who performed significantly better than theoretical chance level (= 50%) on ST level. Due to data corruption, one encoding run was lost for one participant (P07).

The effect of the number of trial repetitions on mean accuracy on individual and group levels was systematically evaluated post hoc.^30^ Answers were decoded anew from a reduced number of trial repetitions following the same approach as described above. Concretely, the accuracies of all consecutive trial combinations for every trial number (1:n trials, where n=[1,2,3,4,5,6,7,8,9,10]) were computed. For example, to obtain the accuracy of eight trials, trial combinations 1-2-3-4-5-6-7-8, 2-3-4-5-6-7-8-9, and 3-4-5-6-7-8-9-10 were used. The effect of number of repetitions on the decoding accuracy at the group level was quantified with Spearman’s rho correlation coefficient. The eﬀect of number of trials was additionally evaluated using information transfer rate (ITR), deﬁned as in Allison, Dunne^52^

$$\mathrm{ITR}=(\log_{2}\;N+P*\log_{2}\;P+(1-P)*\log_{2}(\frac{1-P}{N-1}))*\frac{60}{\tau },$$ (1)

where N is the number of classes, P is the classification accuracy, and τ is the duration of task and rest period, in seconds. Finally mean and standard error of the subjective data (ratings) were computed. Furthermore, the relationship of the ST accuracies and (a) fNIRS-suitability scores, (b) subjective ratings, and (c) localizer t-values within COI were quantified using Pearson’s correlation coefficient.

## 3 Results

### 3.1 Channel Selection

**Fig. 4:**
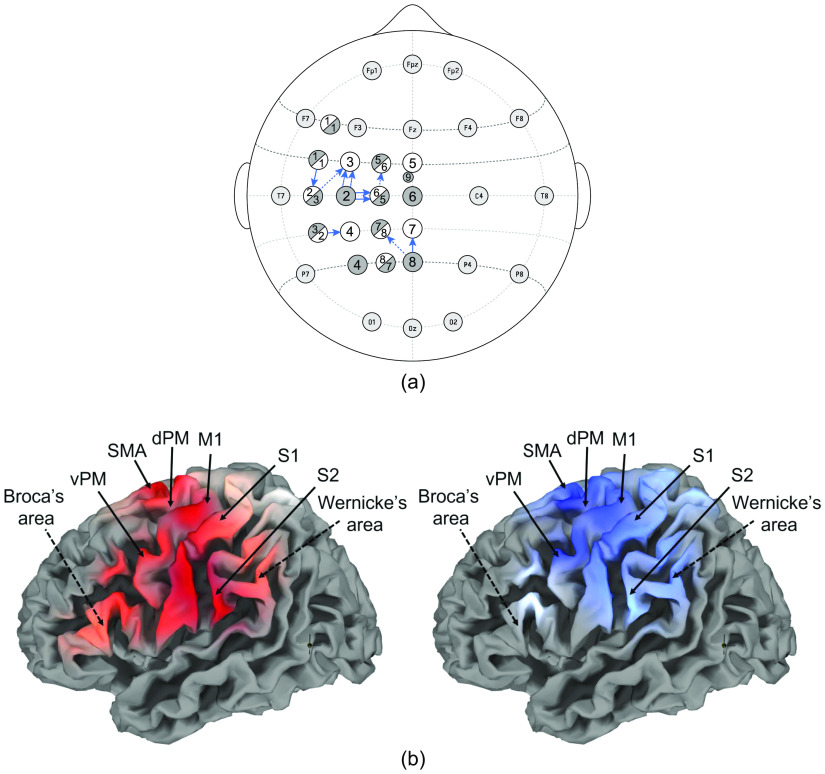
Results of the localizer procedure. (a) Selected COIs for “checkerboard” (arrows) and “rowed” (dashed arrows) setup are marked. Sources (gray) and detectors (white) for both setups are displayed. (b) Topographical maps of HbO (red) and HbR (blue) activation change obtained by group fixed effects analysis (with n=10, Bonferroni-corrected p<0.01) during combined motor-speech imagery task for the “checkerboard” setup. Key speech-imagery (dashed arrows) and right-hand motor-imagery (arrows) areas are marked. Due to the limited spatial resolution of fNIRS, the labeling of the brain regions should be considered an approximation. vPM, ventral premotor area; SMA, supplemental motor area; dPM, dorsal premotor area; M1, primary motor cortex; S1, primary sensory cortex; and S2, secondary somatosensory cortex. Due to an error during the experimental session, a non-ideal setup was chosen for this participant (“checkerboard” instead of “rowed” setup).

Overall, 10 participants took part in the experiment (see Table 1). For all participants, an individual most-informative COI was determined using a localizer procedure described above (see Table 2 for details). Overall, COIs were mostly located above dorsal and ventral premotor area [see Figs. 4(a) and 4(c)]. For two individuals, most reliable activation was detected above superior parietal lobe and precuneus [see Fig. 4(a)]. Generally, the highest t-value was found for changes in HbR concentrations in all participants. The optode-setup choice based on the most-informative COI resulted in the “rowed” setup for 6 out of 10 the participants (see Table 2). The average distance of all COIs was 41.46±6.1  mm as provided by NIRSite (39±3.3  mm for “checkerboard” only; 43.1±7.1  mm for “rowed” only; for more details, see Table S5 in the Supplementary Material).

**Table 2:** Setup choice for each participant based on channel selection.

Participant	Start setup	Most informative channels per setup				p-value of COI	Setup with most informative channel	FNIRS- suitability score
		“Checkerboard”		“Rowed”				
		HbO	HbR	HbO	HbR			
P01	“Rowed”	5-3 (0.96)	4-8 (0.85)	8-8 (1.59)	**8-8 (1.79)**	< .085	“Rowed”	14
P02	“Checkerboard”	2-3 (7.53)	2-3 (10.08)	5-5 (4.68)	**2-3 (10.29)**	< .001	“Rowed”	12
P03	“Checkerboard”	2-6 (34.63)	**2-6 (40.92)**	2-3 (40.09)	2-3 (37.75)	< .001	“Checkerboard”	9
P04	“Rowed”	5-6 (6.99)	9-6 (6.15)	5-5 (10.51)	**5-6 (14.84)**	< .001	“Rowed”	11
P05	“Rowed”	2-6 (2.89)	2-2 (3.42)	2-1 (4.85)	**3-3 (7.31)**	< .001	“Rowed”	10
P06	“Rowed”	2-2 (2.17)	**1-2 (5.46)**	2-2 (5.41)	2-2 (4.86)	< .001	“Checkerboard”	14
P07	“Checkerboard”	1-2 (2.55)	**2-6 (9.77)**	2-1 (4.91)	6-6 (3.57)	< .001	“Checkerboard”	12
P08	“Checkerboard”	1-2 (4.73)	**8-7 (4.79)**	5-7 (3.4)	3-1 (5.21)	< .001	“Rowed”^a^	12
P09	“Checkerboard”	2-6 (6.87)	2-3 (8.14)	2-6 (9.97)	**2-3 (10.24)**	< .001	“Rowed”	13
P10	“Rowed”	3-2 (2.97)	**3-4 (5.04)**	2-1 (3.00)	2-2 (3.56)	< .025	“Checkerboard”	12

Note. The table displays the setup chosen as start setup for the first localizer of each participant. The most informative channels for each setup are reported as well as measured t-values (parentheses) for oxyhemoglobin (HbO) and deoxyhemoglobin (HbR), respectively. The p-value for the most-informative channel across those four was chosen as COI (bold) and determined the chosen optode setup for the encoding runs. FNIRS-suitability scores are reported for comparison.

a Due to a measurement error during the experimental session, a non-ideal setup was chosen for this participant for encoding runs (“checkerboard” instead of “rowed”).

### 3.2 Communication Accuracy

**Fig. 5:**
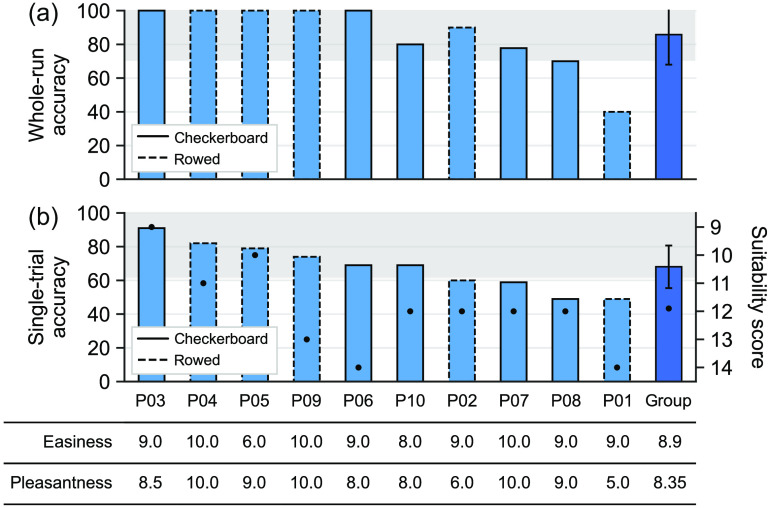
Communication accuracies at individual and group level. Whole-run accuracy (a) and single-trial accuracy (b) are plotted for each participant (sorted by single-trial accuracies) and on group level (x-axis). Standard deviations of group averages are indicated by black lines. Borders of bars represent the individual setup choice. FNIRS-suitability scores are indicated in the single-trial accuracy graph (black dots). The gray areas mark whole-run accuracies ≥70% (a) and single-trial accuracies that were significantly above chance level as determined by a $\chi^{2}$ test (with a corresponding p≤0.05; panel b). Individual easiness/pleasantness ratings of the mental task are provided.

During the sessions, answers could be encoded in real-time using a whole-run online GLM. An average accuracy of 85.78% was obtained across participants with a minimum of 40% (P01) and a maximum of 100% (P03 to P06, P09). All participants except two (P01 and P08) showed an average whole-run accuracy >70% [see Fig. 5(a)]. To speak of a robust communication method, a communication accuracy of more than 70% is considered sufficient in a two-choice paradigm with a sample size equal to 10.^53^^,^^54^

**Fig. 7:**
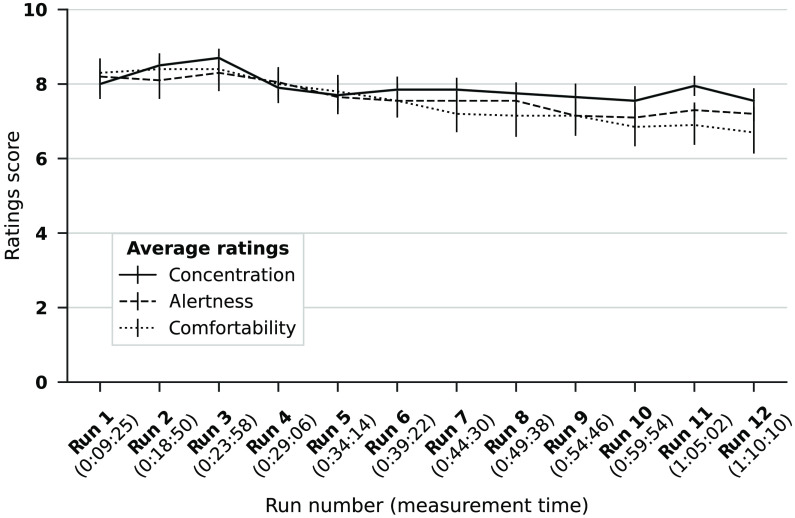
Average subjective ratings. Average ratings of concentration, alertness, and comfortability are plotted for all runs (and corresponding measurement time). Standard errors are indicated by bars.

Using an offline GLM analysis, individual answer trials of participants could be decoded correctly with an average accuracy of 68.09% (theoretical chance level: 50%). ST accuracies varied overall from 49% (P01 and P08) to 91% (P03). In 6 out of 10 participants, ST accuracies were significantly above chance level as calculated with a $\chi^{2}$ test [see Fig. 5(b)]. Average decoding accuracy of the first five trials (true answer encoded) within an encoding run was 67.0±0.15%, whereas it was 69.2%±0.14% for the second half (opposite answer encoded). Average accuracies of trials of “yes” or “no” encodings were 68.4%±0.13% and 67.8%±0.15%, respectively (see Table S4 in the Supplementary Material). Finally, ST accuracies correlated significantly with fNIRS-suitability scores (r=−0.67, p=.03) and localizer t-values within COI (r=0.71, p=0.02). Subjective ratings concerning experienced comfortability, alertness, and concentration (see Fig. 7) did not correlate with ST accuracies. Note that these correlations and their significance should be interpreted with caution as the number of participants was not sufficiently high to draw a firm conclusion.

### 3.3 Effects of Number of Trial Repetitions

**Fig. 6:**
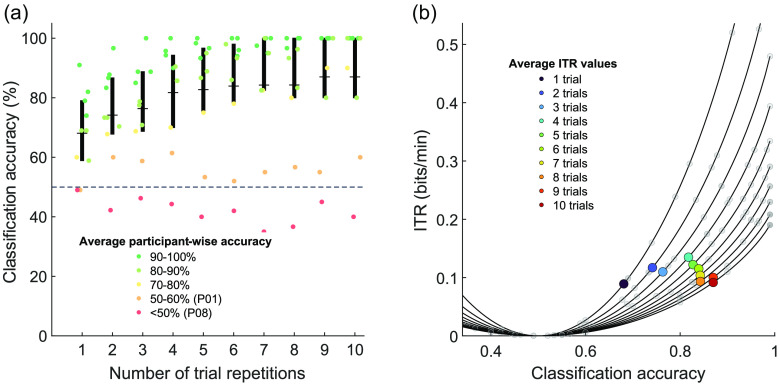
Effect of the number of trial repetitions on decoding accuracy. (a) The box-plot shading depicts classification accuracy for all numbers of repetitions used for decoding: from ten trials to a single trial. Mean values (horizontal lines) and ITR (black bars) are indicated for each number of trials. Participant-wise accuracies (%) are plotted for each number of trial repetitions (colored dots), and average participant-wise accuracy is represented through colors ranging from green to red. The dashed line shows the chance level. The number of trials used to decode each run inﬂuences the decoding process. Mean- and several single-participant accuracies remain above chance level even on a single-trial level. (b) Average (colored markers) and single-participant (gray markers) ITR values (bits/min) for different numbers of trial repetitions (curves) as a function of achieved classiﬁcation accuracies. Lines represent the theoretical values the ITR can take (ranging from 0 to 1) as a function of the number of classes, trial duration and accuracy based on Eq. (1).

The assessment of the effect of the number of trial repetitions on decoding accuracy showed that a mean accuracy >70% could have been achieved already when averaging the data of two trials (μ=74.15%, SD=15.67%, and $M_{x}=73.33\%$) and an accuracy of >80% when considering data of four trials (μ=81.76%, SD=17.69%, and $M_{x}=87.86\%$). The average accuracy reaches the maximum of 87% after nine trial repetitions and then stagnates [see Fig. 6(a)]. A significant positive correlation between the accuracies and the number of repetitions was found as calculated with Spearman’s rho correlation coefficient (ρ=.997, p<0.001). Moreover, the ITR computation indicates that the highest ITR values can be reached, on average, when using four trials (0.13  bits/min) instead of 10 [0.09  bits/min; see Fig. 6(b)].

### 3.4 Subjective Ratings

Subjective ratings of comfortability, alertness, and concentration remained high overall (concentration: μ=7.91±0.95; alertness: μ=7.64±1.14; comfortability: μ=7.53±1.26; see Fig. 7). They ranged from 5 (P02) to 10 (P04) for concentration, 4 (P05) to 8 (P04) for alertness, and 4 (P05/P10) to 10 (P04/P08) for comfortability (see Tables S1–S3 in the Supplementary Material). A decrease of all subject ratings could be observed from the first to last run. The steepest decrease, while still mediocre, was observed in comfortability (from 8.3±1.19 in the first to 6.7±1.79 in the last run). The task was judged as pleasant and easy (pleasantness: μ=8.35±1.61; easiness: μ=8.9±1.14; see Fig. 7). Comparing ratings and accuracy, no obvious trend was observable.

## 4 Discussion

Motor-independent communication through BCIs might improve the quality of life of patients with severe motor paralysis. Though essential in daily life, communication is difficult for these patients. While efforts are made to provide means of communication through brain-based paradigms, complex mental imagery over a prolonged period of time can be exhausting for patients and caregivers alike. A shorter encoding time may result in less fatigue for patients and faster exchange with caregivers about crucial information (i.e., pain indication and basic needs).

Reliance on fNIRS-signal features that can be voluntarily modulated for encoding BCI commands offers a viable addition to established BCI-input modalities, such as EEG and fMRI. Especially the portability of the fNIRS equipment and its easy application highlights the potential of its use by non-experts (e.g., patients’ caretakers) and at the bedside compared to other methods. Successful communication with fNIRS-based BCIs has already been demonstrated in healthy participants (e.g., Refs. 22 and 25) and patients (e.g., Refs. 1 and 37). In previous studies, the speed of information encoding was usually limited by implementing relatively long encoding windows of 6 s^30^ up to 30 s^37^ with many paradigms using periods of 10^1^^,^^2^^,^^25^^–^^29^ to 20 s.^2^^,^^9^^,^^15^^,^^31^ In the present study, participants successfully communicated binary answers through fNIRS signals evoked by performing a differently timed mental-imagery task cued by simple auditory instructions. Participants indicated their choice by performing a combined motor-speech imagery task for ∼2  s only. An individualized localizer procedure together with a combined use of motor and speech imagery facilitated high accuracy. Overall, the paradigm required only a short time of concentration on a relatively easy mental task thus considerably limiting the cognitive effort for BCI users. This makes this communication paradigm ideal for users with limited cognitive resources or cognitive impairments, for example patients or children. The increased efficiency and convenience further emphasize the potential of fNIRS as BCI-input modality.

### 4.1 Encoding Period of 2 s Enables Effective and Convenient Communication

To advance progress towards an efficient and convenient binary fNIRS-BCI, a communication paradigm exploiting temporal fNIRS-signal features and using an encoding time of only 2 s was tested. Whole-run communication accuracies were decoded online. On average, a high online communication accuracy was obtained (μ=85.78%). While communication accuracy varied inter-individually, a sufficient communication accuracy (>70%) was obtained for the vast majority (80%) of the tested healthy participants. Only two participants (P01 and P08) obtained communication accuracies that did not exceed the 70% criterion considered necessary for robust 2-class communication [see Fig. 5(a)].^53^^,^^54^

The data of the communication runs were further analyzed offline to obtain individual ST accuracies and a group mean. Overall, a high ST accuracy (68.09%) was achieved with 6 out of 10 participants showing a significant individual ST accuracy. Expectably, only chance-level ST accuracies were obtained for those two participants for whom insufficient communication accuracies were observed online [see Fig. 5(b)]. By comparison, other studies employing a binary communication approach obtained ST decoding accuracies of 64.4%^36^ and 77.2%.^34^ Communication accuracy obtained when combining several trials reached comparable results when decoded in real-time. One study, for instance, obtained 67% accuracy when combining 20 trials in real-time analysis.^25^ Another study reached 77.4% online accuracy combining three 20 s-trials after training a classifier offline.^39^ Offline analysis of multiple combined trials generally yields better results. A study combining 20 trials of 15 s-encoding windows each obtained an offline whole-run accuracy of 80%.^9^ Accuracy went up as high as 89% in a study by Sitaram et al.^2^ utilizing 20 trials with 10 s-encoding windows per mental imagery task.

Notably, the ITR of the paradigm is low for any number of trial repetitions compared to more advanced communication BCIs.^30^^,^^55^ This is partly due to the length of the pre- and post-window of trials and the small number of answer options, which both yields lower ITRs regardless of accuracy compared to more complex paradigms. The calculation of the ITR was useful in showing the potential for reducing the number of trial repetitions needed for robust communication in some participants. Regardless, the paradigm’s value lies not in increasing the ITR, instead its focus was the decrease of effort for participants through a shorter encoding time. Results indicate that a mental imagery task of 2-s duration is sufficient for most participants to answer binary questions accurately.

Overall, results show that encoding windows as short as 2 s yield comparable accuracies both on an ST and multi-trial level of analysis. Importantly, most participants (7/10) could have reached sufficient accuracy when considering as few as four or even fewer trials as shown during offline analysis, suggesting that speed of communication can be further increased. Note however, that for some individuals (P01, P02, and P08), a larger number of trials encoding one answer or a longer encoding period might have been beneficial and could result in robust communication accuracy (see Fig. 6).

### 4.2 Most Informative Channels over the Prefrontal Motor Areas

It was expected that activation caused by the combined motor-speech imagery would be reflected in channels directly above the prefrontal motor areas. In line with this, the most informative channel was located above these areas in most of the participants (8/10). For two pairs of participants, the same channels were chosen as COI (S2-D3 and S2-D5, respectively, see Table 2). While the combinatory use of the two different optode setups (“checkerboard” and “rowed”) led to a longer experimental session and more effort for the participants (due to performing an additional localizer), it was a pragmatic approach to improve coverage of the fronto-temporo-parietal cortex. Results demonstrated that the COI was found almost equally often in the “checkerboard” and the “rowed” setup (see Table 2, Fig. 3) suggesting that using different setups might be useful. Note, however, that the present study was not intended (and is not suited) to systematically evaluate potential benefits of such an approach. This methodological aspect is currently investigated in a separate study.

Due to an experimenter error, the wrong setup (the “checkerboard” setup) was used during the encoding runs for one participant (P08). The most-informative COI in the ideal (“rowed”) setup (S3-D1) for this participant was actually located above the prefrontal motor areas whereas the used COI (S8-D7) was located more posterior above the parietal cortex. The fact that the premotor area was selected for use in the encoding runs for almost all other participants, suggests that this difference in location could (at least partially) explain why decoding results for P08 were low. Notably, the selected COI of the other participant with chance-level accuracy (S8-D8, P01) was also located more caudally although the signal quality of this participant was generally weak, and the location overlap here might be just coincidental. It should be noted that only one SDC was included in each setup although the use of multiple SDCs per setup would be ideal to account for the heterogeneity in scalp hemodynamics when covering several brain regions.^56^ While this might make application of setups in a natural (non-lab) environment easier, future studies should investigate whether signal quality can be significantly improved through usage of SDCs on multiple sites. Furthermore, a potential research question is whether participants have individual ideal locations for SDCs. If only one SDC is available, it would be ideal to place the SDC accordingly.

### 4.3 On the Benefits of Using a Combined Motor-Speech Imagery Task and the Association of fNIRS Suitability and BCI Performance

The purpose of using a combined motor-speech imagery task was to increase the amount of cortex activated by mental-task performance and therewith to boost the chance to define a promising channel in each participant. After the last encoding run, participants were asked to rate the easiness and pleasantness of the mental task. The task was rated easy and pleasant on average, suggesting that it required only little cognitive effort (see Fig. 7). Since the present study was meant to assess feasibility of shortened encoding times rather than cognitive workload, simple subjective ratings were collected to confirm that the combination of motor and speech imagery was not too demanding for participants. A more detailed systematic evaluation of cognitive workload in relation to the paradigm and imagery could be done in future work. Additionally, one should systematically investigate whether an increased number of answer options could result in high cognitive demand despite short encoding time.

Moreover, participants were asked to rate their current comfortability, concentration, and alertness on a scale between 1 (not at all) and 10 (very) after each run. Ratings of concentration, alertness, and comfortability during the experimental session remained generally high. A small decrease could be observed over runs, particularly for comfortability (see Fig. 7 and Tables S3–S5 in the Supplementary Material). Note that the measurements took place in a darkened room with closed door to improve signal quality and to keep the environment constant across participants. A more natural measurement setting might improve user comfort. To quantify participants’ suitability for measurements, an fNIRS suitability score was determined for each participant by means of a questionnaire filled out prior to the experimental session (see Appendix 2 in the Supplementary Material). ST accuracies correlated negatively with the fNIRS-suitability scores indicating high scores (inferior suitability) can be predictive of limited accuracy [see Fig. 5(b)]. It should be noted that the participant with the lowest accuracy (P01) also had a quite high fNIRS suitability score (14/21 possible points) indicating low fNIRS suitability in terms of hair texture, hair and skin color, and/or head size. While the score offers the possibility to predict a participant’s BCI performance to some degree, obtained communication accuracies also show that participants can still yield great accuracy despite a suboptimal fNIRS-suitability score (e.g., P06). Thus, while the score may be used as an initial judgement method of general fNIRS suitability, it is not the only factor that determines BCI performance. Limited fNIRS suitability might be compensated by other factors, such as motivation or mental-task performance.

### 4.4 Remaining Limitations

The present study has some limitations that should be considered. Coincidentally, only female participants were included; in addition, the number of participants is limited (n=10) although in BCI experiments (where the focus is on the individual level), the current sample size is sufficient to demonstrate feasibility of the paradigm at hand.^2^^–^^4^^,^^7^^,^^9^ A replication of the study, preferably performed in another lab, with a larger number of participants, including male participants would be a valuable extension.

While the overall aim was to design a paradigm aiding everyday use of an fNIRS-based communication BCI, the conditions under which answers were encoded, were kept as constant across participants as possible. However, while a darkened, closed-off room likely aids consistency and signal quality, it does not represent a realistic situation of potential application in patients. Thus, to conclusively proof suitability of fNIRS for everyday use, this BCI-communication method should also be tested in a less controlled environment, i.e., natural environments, as was done recently in a study by Nagels-Coune et al.^27^

Finally, the design of one communication trial resulted in a shorter resting period following a “yes” compared to a “no” answer which could potentially induce a bias. However, this difference did not confound the present results as there was no difference in encoding accuracies between “yes” and “no” answers (see Table S4 in the Supplementary Material).

### 4.5 Future Research Directions

The presented fNIRS communication BCI opens up several new directions for further investigation. First, the present study used a questionnaire score to determine suitability of participants for fNIRS, and even though obtained scores seem to correlate with ST decoding accuracy, this measurement is based on the subjective evaluation of participants’ physical appearance. The presented FNIRS findings suggest that the signal quality observed during localizer runs could also be indicative of attainable communication accuracy. Future studies should investigate whether one could determine fNIRS suitability of a BCI user based on localizer data alone. Second, the chosen combined motor-speech imagery task was thought to be easy in terms of cognitive effort while also increasing the chance to find a suitable COI in all participants. To further improve the signal quality for each participant individually, one could choose an individually selected, optimal task per user which elicits the best possible signal. Finally, results (see Fig. 6) indicate that, at least for certain participants, the number of trials required for robust answer decoding can be reduced. It remains to be tested whether it is possible to predict the number of required trials for an individual to adjust run time accordingly and then gain sufficient accuracy for all tested participants who generally are suitable for fNIRS.

## 5 Conclusion

With increased speed of communication enabled through the shorter encoding time windows, the present study gives proof of concept for convenient, efficient, immediate, and motor-independent communication with healthy human participants. Working memory load for participants is kept at a minimum through the short phase of encoding and the use of a single simple mental imagery task. A relatively easy BCI setup enabled through use of fNIRS-based neuroimaging could make the paradigm a feasible option for use at patients’ bedsides as well. Future studies should focus on tailoring (fNIRS-)BCIs to individual needs and further improve comfortability for the user.

## Code, Data, and Materials Availability

The raw data supporting the conclusions of this article can be found here: https://doi.org/10.34894/XJSOFU. Code for analysis and figures is available here: https://github.com/acv132/2seconds2choiceFNIRSBCI.
