# Supplementary material for: It takes two (seconds): decreasing encoding time for two-choice functional near-infrared spectroscopy brain–computer interface communication
Source: Neurophotonics. 2023 Nov 2;10(4):045005. doi: 10.1117/1.NPh.10.4.045005 (PMC10620514; doi:10.1117/1.NPh.10.4.045005)
Supplement: Supplementary file 1 [file NPh_010_045005_SD001.docx]

10 Supplementary Material

**10.1 Appendix 1: Binary Questions**

| 1. | Do you have a brother? |
| --- | --- |
| 2. | Do you sleep before 12'o clock? |
| 3. | Do you own a car? |
| 4. | Have you been beyond Europe already? |
| 5. | Were you born in Maastricht? |
| 6. | Would ever like to visit the moon? |
| 7. | Do you like sports? |
| 8. | Have you ever driven a motor scooter? |
| 9. | Are you married? |
| 10. | Do you drink coffee in the morning? |
| 11. | Do you like listening to classical music? |
| 12. | Are you older 28 years? |
| 13. | Do you have a driver's licence? |
| 14. | Do you eat pork? |
| 15. | Do you have any sisters? |
| 16. | Is your favorite color red? |
| 17. | Do you like cats? |
| 18. | Do you go on winter holidays? |
| 19. | Do you have children? |
| 20. | Do you watch the news at 8'o clock? |
| 21. | Do you have a smartphone? |
| 22. | Were you born before 1985? |
| 23. | Do you live in Maastricht? |
| 24. | Do you have a dog? |
| 25. | Were you born in the Netherlands? |
| 26. | Do you live in a house? |
| 27. | Did you study in Germany? |
| 28. | Do you like spaghetti? |
| 29. | Do you go on summer holidays? |
| 30. | Do you have a laptop? |
| 31. | Do you miss your hometown? |
| 32. | Did you graduate in Cologne? |
| 33. | Did you visit the school in Magdeburg? |
| 34. | Do you want to stay forever in your current home town? |
| 35. | Did you have a job while going to school? |
| 36. | Do you like the summer season? |
| 37. | Did you graduate in Maastricht? |
| 38. | Have you ever visited USA? |
| 39. | Do you like the colour pink? |
| 40. | Do you like to play football? |
| 41. | Did you immediately find a job after your education? |
| 42. | Do you like the winter season? |
| 43. | Do you like to play volleyball? |
| 44. | Do you like to swim in the sea? |
| 45. | Do you like your hometown? |

## **10.2 Appendix 2: FNIRS Suitability Score Template**

**10.3 Appendix 3: Tables**

**Table 1**

*Concentration Ratings Across Runs*

| Participant | Run | | | | | | | | | | | | Average |
| --- | --- | --- | --- | --- | --- | --- | --- | --- | --- | --- | --- | --- | --- |
|  | 1 | 2 | 3 | 4 | 5 | 6 | 7 | 8 | 9 | 10 | 11 | 12 |  |
| P01 | 6 | 8 | 8 | 6 | 6 | 7 | 7 | 7 | 6 | 7 | 7 | 6 | 6.75 |
| P02 | 8 | 9 | 9 | 7 | 6 | 8 | 7 | 8 | 8 | 5 | 8 | 7 | 7.5 |
| P03 | 8 | 8 | 9 | 9 | 9 | 8.5 | 8.5 | 8.5 | 8.5 | 8.5 | 8.50 | 8.5 | 8.54 |
| P04 | 10 | 10 | 10 | 10 | 10 | 10 | 10 | 10 | 10 | 10 | 10 | 10 | 10 |
| P05 | 6 | 6 | 7 | 6 | 6 | 6 | 6 | 7 | 6 | 7 | 8 | 7 | 6.5 |
| P06 | 9 | 9 | 9 | 8 | 8 | 8 | 8 | 7 | 7 | 7 | 7 | 7 | 7.83 |
| P07 | 9 | 9 | 9 | 9 | 8 | 8 | 8 | 7 | 8 | 8 | 8 | 8 | 8.25 |
| P08 | 7 | 9 | 9 | 8 | 8 | 7 | 8 | 8 | 8 | 8 | 8 | 7 | 7.92 |
| P09 | 9 | 9 | 9 | 9 | 9 | 9 | 8 | 8 | 8 | 8 | 8 | 8 | 8.5 |
| P10 | 8 | 8 | 8 | 7 | 7 | 7 | 8 | 7 | 7 | 7 | 7 | 7 | 7.33 |
| Mean | 8 | 8.5 | 8.7 | 7.9 | 7.7 | 7.85 | 7.85 | 7.75 | 7.65 | 7.55 | 7.95 | 7.55 | 7.91 |

**Table 2**

*Alertness Ratings Across Runs*

| Participant | Run | | | | | | | | | | | | Average | |  |
| --- | --- | --- | --- | --- | --- | --- | --- | --- | --- | --- | --- | --- | --- | --- | --- |
|  | 1 | 2 | 3 | 4 | 5 | 6 | 7 | 8 | 9 | 10 | 11 | 12 | |  | |
| P01 | 9 | 8 | 8 | 7 | 6 | 7 | 7 | 7 | 7 | 7 | 7 | 7 | | 7.25 | |
| P02 | 8 | 7 | 8 | 8 | 8 | 8 | 8 | 9 | 7 | 6 | 8 | 8 | | 7.75 | |
| P03 | 8 | 8 | 9 | 8.5 | 8.5 | 8.5 | 8.5 | 8.5 | 8.5 | 8 | 8 | 8 | | 8.33 | |
| P04 | 10 | 10 | 10 | 10 | 9 | 9 | 9 | 8 | 8 | 8 | 8 | 8 | | 8.92 | |
| P05 | 4 | 4 | 4 | 5 | 4 | 4 | 4 | 4 | 4 | 5 | 6 | 6 | | 4.5 | |
| P06 | 9 | 9 | 9 | 8 | 8 | 8 | 8 | 8 | 7 | 7 | 7 | 7 | | 7.92 | |
| P07 | 9 | 9 | 9 | 9 | 9 | 9 | 9 | 8 | 8 | 8 | 7 | 7 | | 8.42 | |
| P08 | 8 | 9 | 9 | 9 | 8 | 8 | 7 | 8 | 7 | 8 | 8 | 7 | | 8 | |
| P09 | 9 | 9 | 9 | 8 | 8 | 7 | 7 | 7 | 7 | 7 | 7 | 7 | | 7.67 | |
| P10 | 8 | 8 | 8 | 8 | 8 | 7 | 8 | 8 | 8 | 7 | 7 | 7 | | 7.67 | |
| Mean | 8.2 | 8.1 | 8.3 | 8.05 | 7.65 | 7.55 | 7.55 | 7.55 | 7.15 | 7.1 | 7.3 | 7.2 | | 7.64 | |

**Table 3**

*Comfortability Ratings Across Runs*

| Participant | Run | | | | | | | | | | | | Average | |
| --- | --- | --- | --- | --- | --- | --- | --- | --- | --- | --- | --- | --- | --- | --- |
|  | 1 | 2 | 3 | 4 | 5 | 6 | 7 | 8 | 9 | 10 | 11 | 12 | |  |
| P01 | 7 | 7 | 8 | 8 | 8 | 8 | 8 | 8 | 8 | 8 | 8 | 8 | | 7.83 |
| P02 | 7 | 8 | 8 | 7 | 7 | 8 | 7 | 9 | 9 | 7 | 8 | 8 | | 7.75 |
| P03 | 9 | 9 | 9 | 9 | 9 | 9 | 8.5 | 8.5 | 7.5 | 7 | 6.5 | 6 | | 8.17 |
| P04 | 10 | 10 | 10 | 10 | 10 | 10 | 10 | 10 | 10 | 10 | 10 | 10 | | 10 |
| P05 | 8 | 7 | 7 | 6 | 5 | 5 | 4 | 4 | 4 | 4 | 4 | 4 | | 5.17 |
| P06 | 9 | 8 | 8 | 8 | 8 | 8 | 7 | 7 | 7 | 7 | 7 | 6 | | 7.5 |
| P07 | 9 | 9 | 9 | 8 | 8 | 7 | 7 | 6 | 8 | 8 | 8 | 8 | | 7.92 |
| P08 | 6 | 10 | 10 | 10 | 9 | 8 | 8 | 8 | 7 | 7 | 7 | 7 | | 8.08 |
| P09 | 9 | 8 | 8 | 8 | 8 | 7 | 7 | 6 | 6 | 6 | 6 | 6 | | 7.08 |
| P10 | 9 | 8 | 7 | 6 | 6 | 5.5 | 5.5 | 5 | 5 | 4.5 | 4.5 | 4 | | 5.83 |
| Mean | 8.3 | 8.4 | 8.4 | 8 | 7.8 | 7.55 | 7.2 | 7.15 | 7.15 | 6.85 | 6.9 | 6.7 | | 7.53 |

**Table 4**

*Single-trial accuracies and χ^2^ values*

|  | Single-trial accuracy | | | | | χ^2^ (corresponding to total) | | |
| --- | --- | --- | --- | --- | --- | --- | --- | --- |
| Participants | Trials | | | Answers | | Value | p-value | Effect size |
|  | Total | 1. Half | 2. Half | Yes | No |  |  |  |
| P01 | 0.49 | 0.48 | 0.5 | 0.48 | 0.5 | 0.08 | 0.99 | - |
| P02 | 0.6 | 0.56 | 0.64 | 0.64 | 0.56 | 4.64 | 0.2 | - |
| P03 | 0.91 | 0.92 | 0.9 | 0.9 | 0.92 | 67.28 | < .001 | 0.82 |
| P04 | 0.82 | 0.82 | 0.82 | 0.84 | 0.8 | 41.12 | < .001 | 0.64 |
| P05 | 0.79 | 0.74 | 0.84 | 0.76 | 0.82 | 34 | < .001 | 0.58 |
| P06 | 0.69 | 0.66 | 0.72 | 0.68 | 0.7 | 14.48 | < .001 | 0.38 |
| P07 | 0.59 | 0.6 | 0.58 | 0.58 | 0.6 | 3.21 | 0.36 | - |
| P08 | 0.49 | 0.48 | 0.5 | 0.52 | 0.46 | 0.4 | 0.94 | - |
| P09 | 0.74 | 0.78 | 0.7 | 0.72 | 0.76 | 23.2 | < .001 | 0.48 |
| P10 | 0.69 | 0.66 | 0.72 | 0.72 | 0.66 | 14.8 | < .001 | 0.38 |
| Mean | 0.68 | 0.67 | 0.69 | 0.68 | 0.68 |  |  |  |

**Table 5**

*Distances of channels*

| Setup | Sources | Detectors | | | | | | | |
| --- | --- | --- | --- | --- | --- | --- | --- | --- | --- |
|  |  | D1 | D2 | D3 | D4 | D5 | D6 | D7 | D8 |
| checkerboard | S1 | 34.1 | **34** | 37 | 0 | 0 | 0 | 0 | 0 |
|  | S2 | 0 | 40.7 | 37.6 | 36.7 | 0 | **40.9** | 0 | 0 |
|  | S3 | 0 | 33.6 | 0 | **40.1** | 0 | 0 | 0 | 0 |
|  | S4 | 0 | 0 | 0 | 36.4 | 0 | 0 | 0 | 33 |
|  | S5 | 0 | 0 | 37.2 | 0 | 37 | 39.3 | 0 | 0 |
|  | S6 | 0 | 0 | 0 | 0 | 40.1 | 41 | 39.9 | 0 |
|  | S7 | 0 | 0 | 0 | 40.3 | 0 | 38.9 | 40.2 | 38.5 |
|  | S8 | 0 | 0 | 0 | 0 | 0 | 0 | 39.7 | 33.1 |
|  | S9 | 0 | 0 | 0 | 0 | 12.2 | 48.9 | 0 | 0 |
| rowed | S1 | 34.1 | 0 | 49.7 | 0 | 0 | 0 | 0 | 0 |
|  | S2 | 54.1 | 51.7 | **37.6** | 36.7 | 0 | 54.3 | 0 | 56.7 |
|  | S3 | 34 | 33.6 | **51.4** | 55.3 | 0 | 0 | 0 | 0 |
|  | S4 | 0 | 50.6 | 0 | 36.4 | 0 | 0 | 0 | 52.6 |
|  | S5 | 0 | 0 | 55.2 | 54.2 | 55.4 | **39.3** | 56.7 | 38.9 |
|  | S6 | 0 | 0 | 0 | 0 | 40.1 | 55.8 | 39.9 | 56.5 |
|  | S7 | 0 | 0 | 0 | 52 | 0 | 0 | 53.8 | 38.5 |
|  | S8 | 0 | 0 | 0 | 0 | 0 | 0 | **39.7** | **53.1** |
|  | S9 | 0 | 0 | 0 | 0 | 12.2 | 40.3 | 0 | 0 |

*Note.* Used channels are marked through a shaded area. Distances between sources (“S”) and detectors (“D”) are displayed in millimeters for both setups. Distances of channels of interest are printed in bold.
